# Bismuthene Under Cover: Graphene Intercalation of a Large Gap Quantum Spin Hall Insulator

**DOI:** 10.1002/adma.202502412

**Published:** 2025-05-20

**Authors:** Lukas Gehrig, Cedric Schmitt, Jonas Erhardt, Bing Liu, Tim Wagner, Martin Kamp, Simon Moser, Ralph Claessen

**Affiliations:** ^1^ Physikalisches Institut Universität Würzburg D‐97074 Würzburg Germany; ^2^ Würzburg‐Dresden Cluster of Excellence ct.qmat Universität Würzburg D‐97074 Würzburg Germany; ^3^ Physikalisches Institut and Röntgen Center for Complex Material Systems D‐97074 Würzburg Germany

**Keywords:** bismuthene, 2D material, graphene intercalation, quantum spin hall insulator, topological insulator

## Abstract

The quantum spin Hall insulator bismuthene, a two‐third monolayer of bismuth on SiC(0001), is distinguished by helical metallic edge states that are protected by a groundbreaking 800 meV topological gap, making it ideal for room temperature applications. This massive gap inversion arises from a unique synergy between flat honeycomb structure, strong spin orbit coupling, and an orbital filtering effect that is mediated by the substrate. However, the rapid oxidation of bismuthene in air has severely hindered the development of applications, so far confining experiments to ultra‐high vacuum conditions. Intercalating bismuthene between SiC and a protective sheet of graphene, this barrier is successfully overcome. As demonstrated by scanning tunneling microscopy and photoemission spectroscopy, graphene intercalation preserves the structural and topological integrity of bismuthene, while effectively shielding it from oxidation in air. Hereby, hydrogen is identified as the critical process gas that was missing in previous bismuth intercalation attempts. These findings facilitate ex‐situ experiments and pave the way for the development of bismuthene based devices, signaling a significant step forward in the development of next‐generation technologies.

## Graphical abstract

Bismuthene, a quantum spin Hall insulator with a massive 800 meV topological gap, holds great potential for room‐temperature devices but rapidly degrades in air. This study demonstrates that intercalating bismuthene between SiC and a graphene sheet preserves its structure and topology while preventing oxidation, enabling ex‐situ experiments and advancing the fabrication of bismuthene‐based devices.

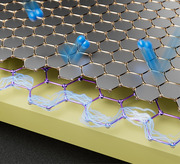

## 1 Introduction

Quantum spin Hall insulators (QSHIs) represent a groundbreaking class of quantum materials, characterized by the quantum spin Hall effect (QSHE).^[^ ^1^, ^2^ ^]^ These 2D materials have garnered significant interest due to their spin‐momentum locked metallic edge states,^[^ ^3^ ^]^ which enable dissipationless electron transport. Such a unique property has profound implications for novel electronic^[^ ^4^, ^5^ ^]^ and spintronic devices.^[^ ^6^, ^7^ ^]^ Additionally, the edge states of QSHIs may host Majorana fermions, positioning these materials as promising candidates for quantum computing.^[^ ^8^, ^9^, ^10^ ^]^

For QSHIs to serve in viable device applications, the spin‐orbit inverted topological band gap *E*_gap_ must substantially surpass thermal broadening, on the order of *E*_gap_ ⩾ 3.5 *k*_*B*_ *T*, implying approximately 90 meV at room‐temperature (RT).^[^ ^11^ ^]^ This requirement excludes graphene, the material initially predicted to exhibit the QSHE,^[^ ^1^ ^]^ due to its minuscule topological band gap of less than 50μeV.^[^ ^12^ ^]^ Building on the honeycomb motif, researchers have substituted carbon atoms in graphene‐like structures with heavier elements, thereby drastically enhancing spin‐orbit coupling (SOC) and increasing the gap.^[^ ^13^ ^]^ A notable breakthrough in this area of 2D “Xene”‐materials is the discovery of bismuthene, a structural twin of graphene, realized as a 2/3 monolayer of bismuth on SiC(0001) (i.e., two bismuth per three terminating silicon atoms) forming a $\left(\sqrt{3}\times \sqrt{3}\right)R30^{\circ }$ superstructure with respect to the SiC surface unit cell.^[^ ^14^, ^15^ ^]^ Bismuthene's remarkable topological band gap of *E*_gap_ ∼ 800 meV still remains the largest reported for any 2D topological insulator to date.

Despite this success, bismuthene's susceptibility to oxidation under ambient conditions poses a significant challenge, limiting its experimental use to ultra‐high vacuum (UHV) environments. To mitigate this problem, metal intercalation underneath a protective sheet of graphene^[^ ^16^, ^17^, ^18^, ^19^, ^20^ ^]^ may prevent oxidation while preserving the material's topological properties, as we recently demonstrated for the QSHI indenene, i.e., a *full* atomic monolayer of indium on SiC(0001) with a 120 meV gap.^[^ ^21^ ^]^ Although bismuth intercalation has been attempted, conventional methods have thus far yielded only trivial bismuth films. These include a fully covered yet metallic structure and a 1/3‐covered insulating structure that remains topologically trivial.^[^ ^22^, ^23^, ^24^ ^]^ Achieving intercalation of the topologically nontrivial 2/3 monolayer bismuthene phase, however, has proven more technologically challenging and has remained elusive. In this work, we address this challenge and introduce a robust protocol for controlling the intercalation process between 1/3 and the full bismuth monolayers, and successfully stabilize the 2/3 monolayer QSHI bismuthene phase beneath a single graphene sheet via an additional hydrogen annealing step. We demonstrate that this graphene layer effectively protects the intercalant from oxidation when exposed to controlled amounts of oxygen and air, while fully preserving its topologically non‐trivial character.

## 2 Results and Discussion

**Figure 1:**
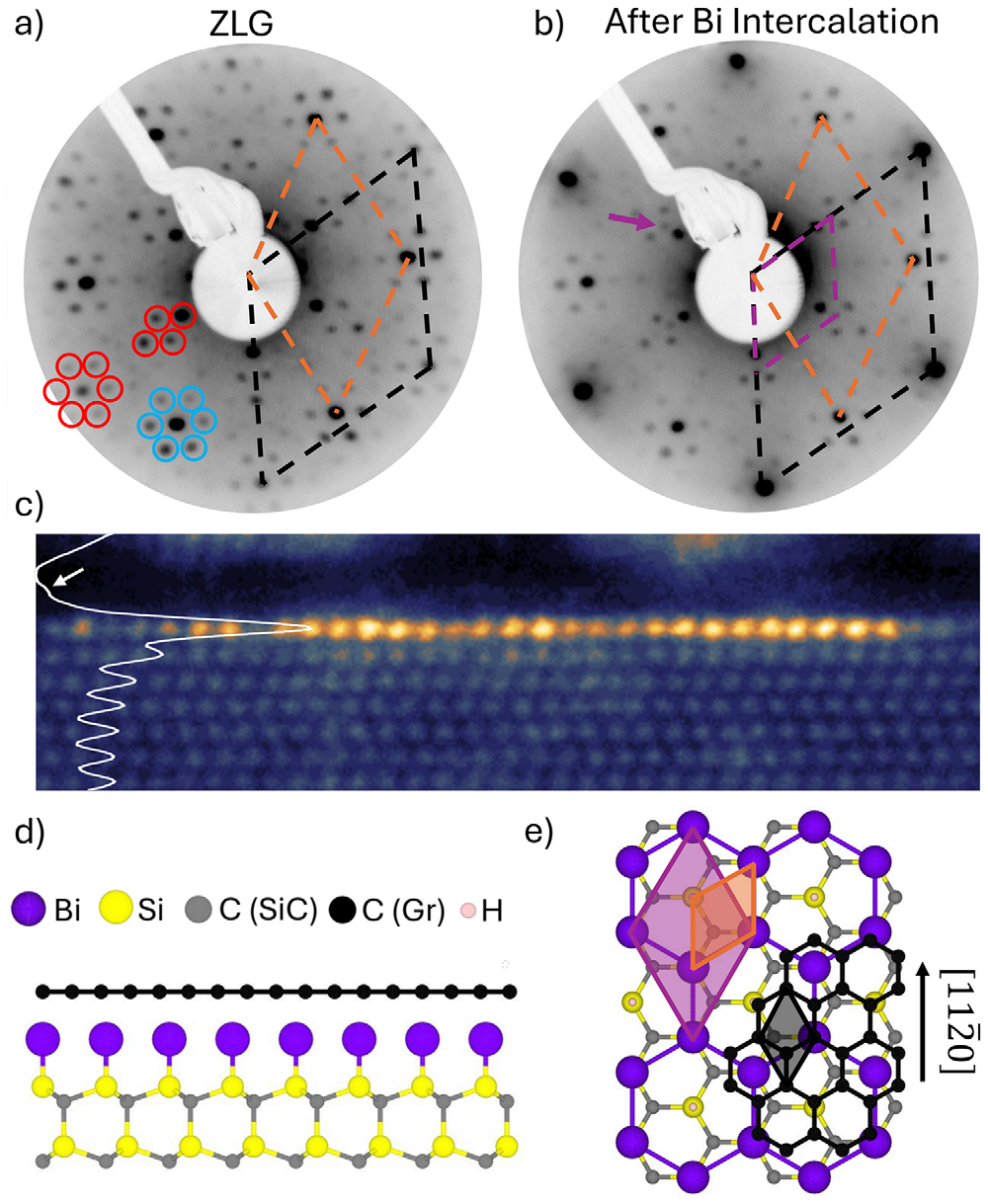
Structural characterization of intercalated bismuthene. LEED taken at 60 eV before a) and after b) bismuthene intercalation. The black, orange and purple contours denote the LEED spots of graphene, SiC and bismuthene, respectively. The additional bismuthene spot is marked by a purple arrow. The spots marked by red and blue circles correspond to the $\left(6\sqrt{3}\times 6\sqrt{3}\right)R30^{\circ }$ and quasi‐(6 × 6) periodicity of ZLG, respectively. c) Cross‐sectional TEM of intercalated bismuthene projecting along the $\left[11\bar{2}0\right]$ direction shows an atomically thin layer of bismuth between SiC and graphene. A shoulder (white arrow) in the horizontally integrated line profile (white line) shows the presence of the graphene sheet. Side view d) and top view e) of the ball and stick model of intercalated bismuthene. The black, orange and purple contours denote the primitive unit cells of graphene, SiC and bismuthene, respectively. By comparing the unit cells, it is evident that bismuthene is a $\left(\sqrt{3}\times \sqrt{3}\right)R30^{\circ }$ superstructure with respect to SiC.

To produce bismuthene intercalated graphene, nitrogen‐doped 4H‐SiC substrates were first dry‐etched in a flowing hydrogen atmosphere of 950 mbar, following the procedure outlined by Glass et al.^[^ ^25^ ^]^ Subsequently, the substrates were annealed at 1100°C in UHV for 15 min to epitaxially grow zero‐layer graphene (ZLG), as previously reported by Riedl et al. in ref. [26]. The resulting low‐energy electron diffraction (LEED) pattern, shown in **Figure** **1**a, reveals the primitive periodicities of both the SiC(0001) surface and graphene, with their respective reciprocal unit cells outlined by orange and black contours. Furthermore, additional LEED peaks, highlighted by red and blue circles, correspond to the characteristic $\left(6\sqrt{3}\times 6\sqrt{3}\right)R30^{\circ }$ and quasi‐(6 × 6) periodicity associated with the ZLG superstructure.^[^ ^26^ ^]^

After safe UHV transfer to our molecular beam epitaxy (MBE) system (Octoplus 300, Dr. Eberl MBE‐Komponenten GmbH), bismuth (99.9999% purity) was deposited from a Knudsen cell held at 550°C. After a one hour bismuth deposition step at room temperature, the sample was subsequently annealed at 350°C for 15 min to achieve bismuth intercalation. For best intercalation results, deposition and annealing were repeated two times, followed by a desorption step at 850°C for 20 min. A subsequent annealing step at 550°C in a 950 mbar standing hydrogen atmosphere for 60 min was crucial for achieving the desired bismuthene phase with 2/3 monolayer coverage. This new process saturates 1/3 of the silicon dangling bonds at the center of the honeycomb structure critical for bismuthene formation, a result that could not be obtained through standard annealing in UHV or argon (see Supplemental Material).

The corresponding LEED pattern in Figure 1b now shows a marked increase in the intensity of the primitive graphene spots, compared to the ZLG phase in Figure 1a. This indicates the decoupling of ZLG from the SiC substrate and the formation of quasi‐freestanding monolayer graphene (QFMG), in analogy to observations in previous graphene intercalation studies.^[^ ^21^, ^27^ ^]^

In addition, the emergence of new LEED spots (purple arrow) after intercalation signifies the formation of a $\left(\sqrt{3}\times \sqrt{3}\right)R30^{\circ }$ bismuth superstructure outlined in purple. Cross‐sectional scanning transmission electron microscopy (STEM) images, shown in Figure 1c, reveal the intercalated bismuth atoms to be indeed confined to a single atomic sheet, while the graphene layer is barely visible in the shoulder of the integrated line profile (white line) due to the low contrast of carbon compared to the heavy bismuth atoms.^[^ ^21^, ^28^ ^]^ The STEM images also confirm that the intercalated bismuth atoms adsorb right atop the silicon atoms, i.e., at the T1 position of the SiC(0001) substrate.

The combined LEED and STEM observations are consistent with a 2/3 monolayer bismuth phase with bismuth occupying the T1 site directly above Si of the first SiC(0001) layer, as reported by Reis et al.,^[^ ^14^ ^]^ but not with the reported 1/3 monolayer phase of previous studies, where bismuth is assumed to adsorb between the Si atoms.^[^ ^22^, ^23^, ^24^ ^]^ Therefore, we conclude the successful intercalation of bismuthene, proposing a structural model as presented in Figure 1d,e.

**Figure 2:**
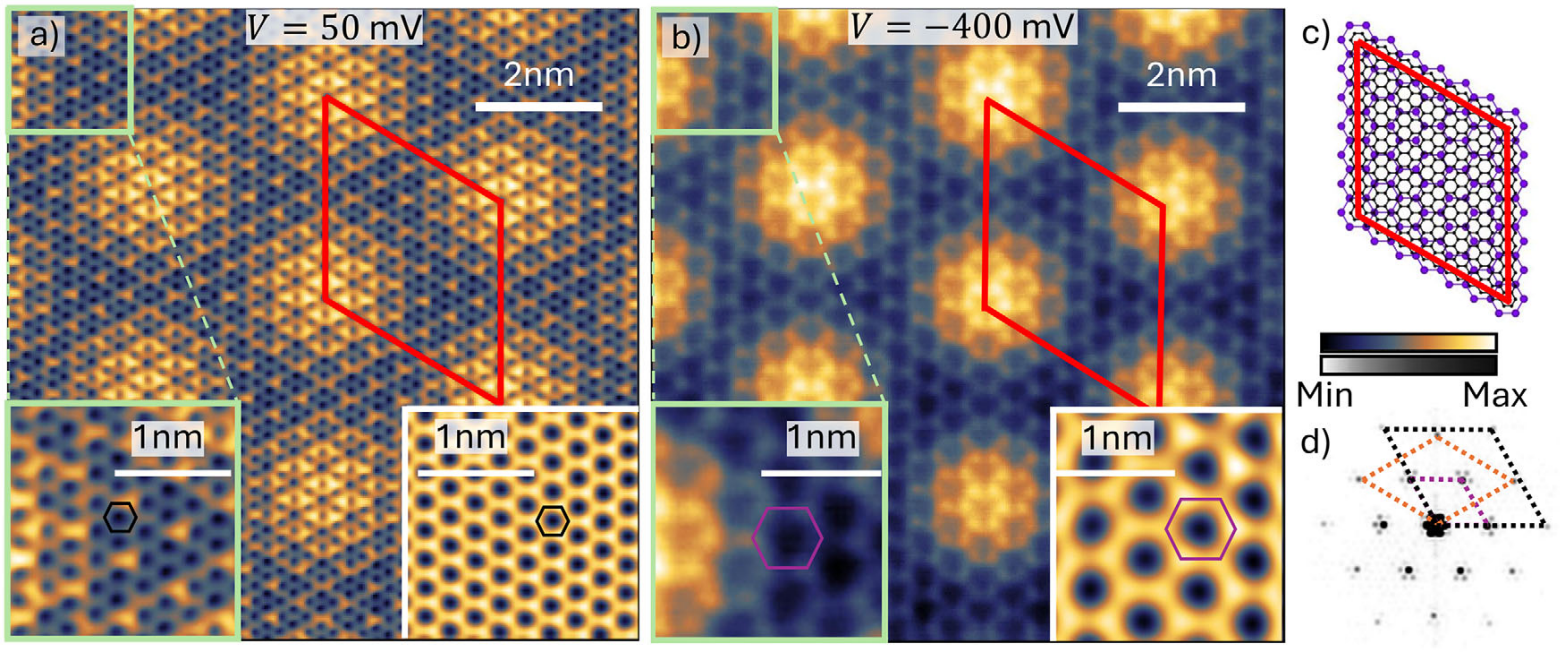
STM of intercalated bismuthene. Constant current STM images measured with bias voltages and tunneling currents of a) 50 mV, 200 pA and b) −400 mV, 180 pA, optimized to resolve the graphene and bismuthene lattices, respectively. Zoom‐ins to the data are shown in the left insets. Corresponding Fourier filtered images are shown in the right inset. c) Moiré unit cell (red contour) arising from the different periodicities of graphene and bismuthene. d) Fast Fourier Transform of the image in b). The spots corresponding to graphene/SiC/bismuthene are linked by black/orange/purple dashed lines to mark the associated Brillouin zones, respectively.

**Figure 3:**
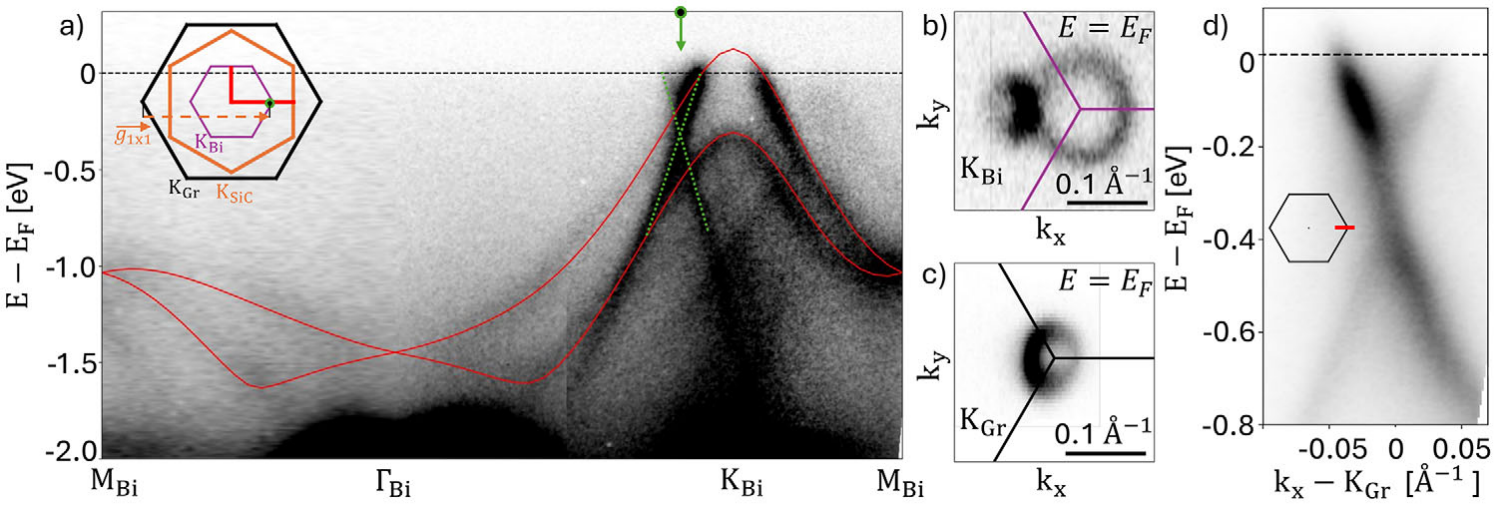
Band structure of intercalated bismuthene. a) *M*_Bi_Γ*K*_Bi_ *M*_Bi_ high‐symmetry band dispersion of intercalated bismuthene measured at room temperature with 21.2 eV (He Iα) photon energy. A graphene replica due to photoelectrons scattering off the (1 × 1) lattice potential of the SiC substrate is also visible (green dashed lines). DFT calculations reproduced from^[^ ^14^ ^]^ are indicated in red. Fermi surfaces around the K‐Points of bismuthene and graphene are shown in b) and c). Panel d) highlights the Dirac crossing of the graphene π‐bands. The *k*_*x*_‐axis points along the red line in the inset.

To determine the relative alignment and interaction between bismuthene and graphene, we present constant current scanning tunneling microscopy (STM) data of a representative 10 × 10 nm^2^ sample region in **Figure** **2**. Figure 2a was measured at a bias voltage of 50 mV, where the bismuthene band structure is gapped, and STM is mostly susceptible to the density of states (DOS) of the graphene overlayer. In contrast, Figure 2b was measured at −400 mV bias voltage, where STM probes mostly the DOS of the bismuthene bulk bands, while the graphene DOS at the Dirac point is suppressed, as we confirm later by ARPES in **Figure** **3**. Via the bias voltage, we thus selectively uncover either the graphene overlayer or the bismuthene intercalant, as seen more clearly in the magnified insets on the left.^[^ ^21^ ^]^ Further, both scans reveal the periodic Moiré pattern formed by the incommensurate bismuthene/SiC(0001) and graphene systems, whose lattice constant of approximately *a*_Moiré_ = 3.225 nm corresponds to roughly 6 *a*_Bi_ or 13 *a*_Gr_.

A fast Fourier transform (FFT) of Figure 2b is shown in Figure 2d and further corroborates this analysis. Similar to the LEED pattern of intercalated bismuthene in Figure 1b, the FFT reveals diffraction spots corresponding, respectively, to the SiC, the graphene, and the bismuthene lattices, marked by orange, black, and purple reciprocal unit cells. By selectively filtering the raw STM data for the spatial frequencies of each lattice, we obtain the clean graphene and bismuthene signatures shown in the right insets, yielding a Moiré unit cell according to Figure 2c. With the structure of intercalated bismuthene now established, we turn our attention to its electronic properties. Room temperature angle‐resolved photoemission spectroscopy (ARPES) measured with He Iα radiation along the *M*_Bi_Γ*K*_Bi_ *M*_Bi_ high‐symmetry path of bismuthene are presented in Figure 3a. The distinctive *p*_*x*_/*p*_*y*_ low‐energy band structure, characteristic of pristine bismuthene,^[^ ^14^ ^]^ is clearly observed and matches the density functional theory (DFT) calculations (red lines) for the pristine phase.^[^ ^13^ ^]^ While a pocket in the Fermi surface of Figure 3b) indicates significant hole doping of (9.2 ± 2.4) × 10^12^ cm^−2^, the presence of a Rashba band splitting at *K*_Bi_ suggests graphene and bismuthene to remain electronically decoupled. In contrast, the characteristic Dirac crossing of graphene's π‐bands shown in Figure 3d is shifted to about –300 meV, indicating significant electron doping of (4.4 ± 1.9) × 10^12^ cm^−2^ extracted from graphene's Fermi surface in Figure 3c. While this electron doping accounts only for about half of bismuthene's holes, the remaining hole concentration is likely contributed by the substrate, similar to the case of indenene.^[^ ^21^ ^]^

Graphene's characteristic *horseshoe* and *dark corridor* features, arising from the honeycomb lattice structure factor, are shown in Figure 3c.^[^ ^29^, ^30^, ^31^ ^]^ Upon closer inspection, we observe a flipped replica of this horseshoe close to *K*_Bi_ in Figure 3b, producing the linear bands (green dashed lines) in the ARPES bandstructure of Figure 3a. As sketched in the inset, this Umklapp arises from graphene photoelectrons scattering off the (1 × 1) lattice potential of the SiC substrate,^[^ ^32^ ^]^ as indicated by the Umklapp vector $g_{1\times 1}$ in Figure 3a.

**Figure 4:**
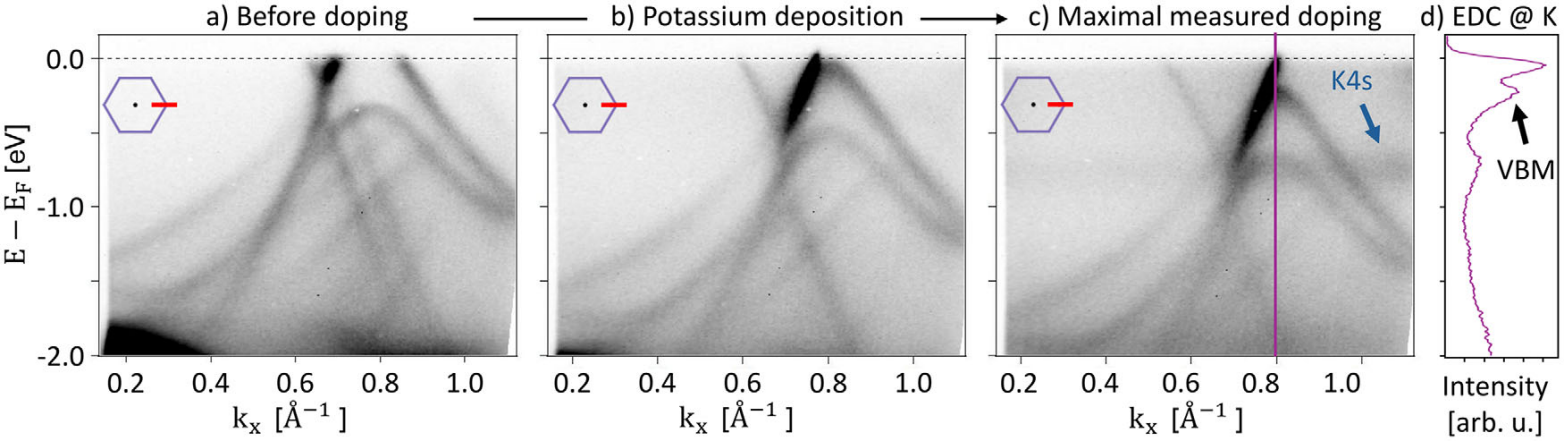
Potassium doping of intercalated bismuthene. a–c) ARPES at bismuthene K point for different levels of potassium doping, measured at 50 K with a photon energy of 21.2 eV (He Iα). d) Doping saturates with bismuthene's valence band maximum (VBM) at about −230 meV, showing intercalated bismuthene to conserve its topological gap. A non‐dispersive band observed at approximately −760 meV in panel c) (blue arrow) is attributed to the 4*s* electrons of potassium.

As the hole doping of bismuthene prevents us from an extraction of its topological gap, we use n‐doping via deposition of potassium while monitoring the valence band around *K*_Bi_ by ARPES.^[^ ^21^ ^]^ As shown in **Figure** **4**, this leads to a gradual filling of graphene and bismuthene with electrons, fully recovering bismuthene's valence band until its maximum saturates at about 230 meV below the Fermi energy in Figure 4c. Although this doping experiment is incapable to resolve the bismuthene band gap in its entirety, the valence band saturation still provides an experimental lower limit of *E*_gap_ ⩾ 230 meV, still sufficiently large as compared to thermal broadening effects at room temperature,^[^ ^11^ ^]^ and likely just an extremely conservative understatement of the real bulk gap *E*_gap_ ∼ 800 meV.

**Figure 5:**
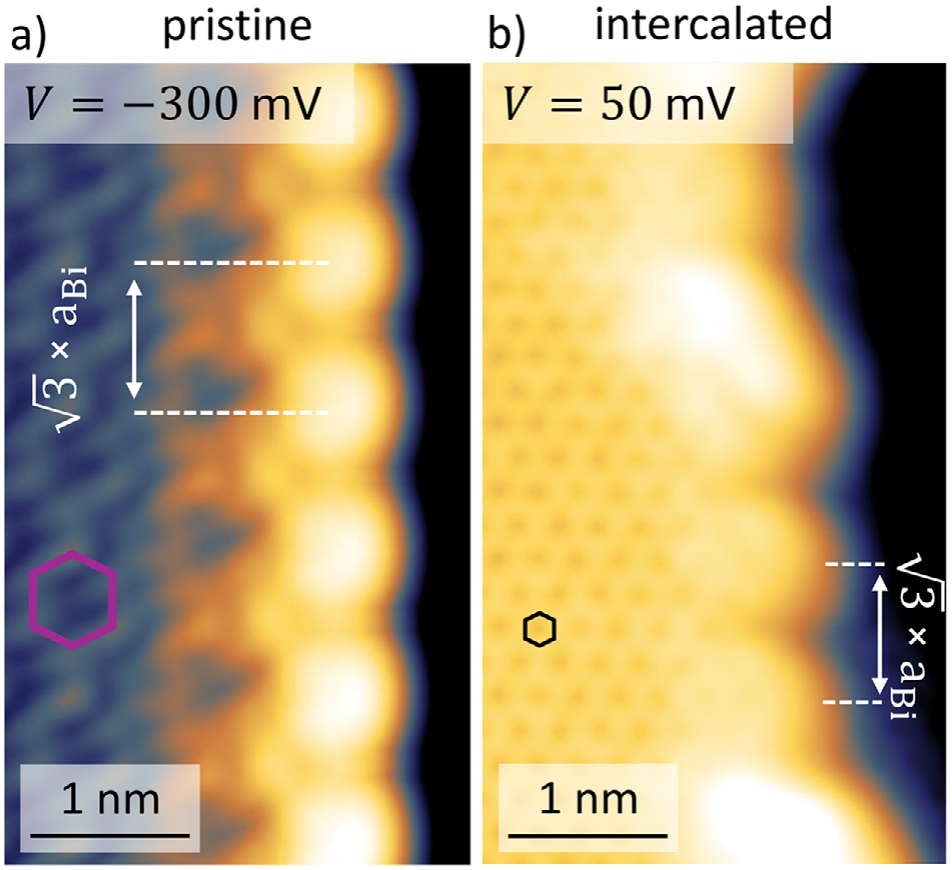
a) Constant current STM images taken at bias voltage of −300 mV at a SiC step edge shows modulation, arising from topological edge states of pristine bismuthene. b) Intercalated bismuthene shows similar modulation at a bias voltage of 50 mV at a SiC step edge. This bias voltage corresponds to the same energy position as in a), accounting for the effective p‐doping of the intercalated variant. A purple and a black hexagon marks the honeycomb of bismuthene and graphene, respectively.

As a consequence of the topological bulk‐boundary correspondence, pristine bismuthene displays metallic edge states filling the substantial band gap. The STM image of **Figure** **5**a displays such a situation at the armchair termination of the bismuthene film at a SiC terrace step, reflected by the enhanced tunneling signal and a characteristic $\sqrt{3}$ periodicity.^[^ ^33^ ^]^ Quite remarkably, we observe identical behavior (enhanced signal and $\sqrt{3}$ modulation) in the armchair‐edges of intercalated bismuthene (see Figure 5b), strongly suggesting the conservation of the topological edge states despite the graphene overlayer.

**Figure 6:**
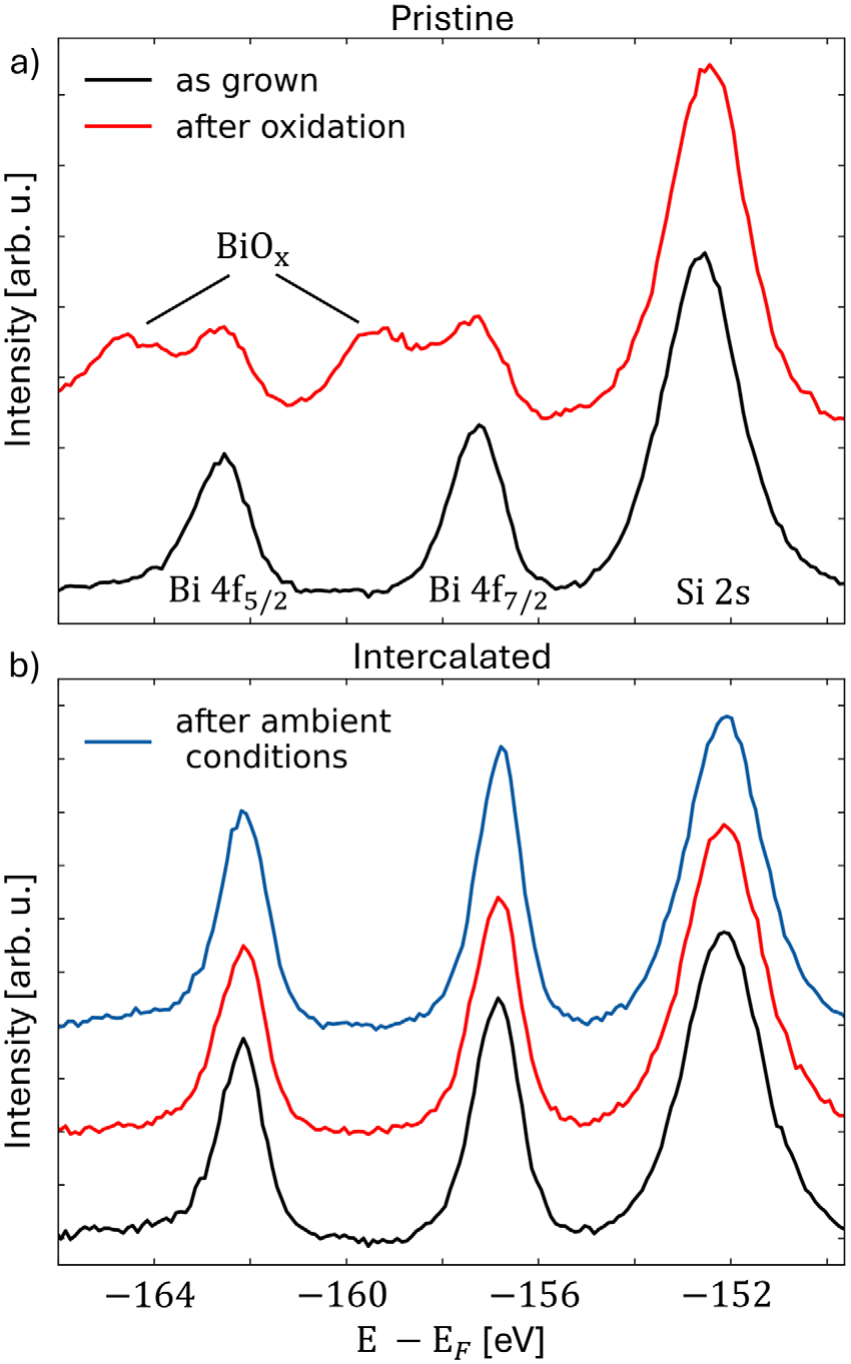
Resilience of intercalated bismuthene in air. XPS spectra of the Bi 4*f* and Si 2*s* core level peaks of a) pristine and b) intercalated bismuthene measured with Al Kα radiation. While pristine bismuthene oxidizes and exhibits XPS satellites upon exposure to oxygen, intercalated bismuthene remains stable even upon exposure to air.

Having confirmed the topological band structure and edge states of intercalated bismuthene, let us finally turn to its robustness against oxidation in ambient conditions. To test this, we exposed both pristine and intercalated bismuthene to ∼2 × 10^−5^ mbar oxygen for 30 minutes and subsequently recorded the X‐ray photoelectron spectroscopy (XPS) spectra of the Bi 4*f* and Si 2*s* core levels. The background‐subtracted and normalized spectra are shown in **Figure** **6**a,b, respectively. In the pristine (uncapped) sample, oxygen exposure introduces two additional Bi 4*f* satellite peaks compared to the as‐grown film, indicating significant oxidation of the topmost bismuth layer. In stark contrast, the Bi 4*f* signature as well as the ARPES signature (see Supporting Information) of intercalated (graphene‐capped) bismuthene remains unchanged even after exposure to oxygen and several days in ambient air, demonstrating the excellent protective properties of graphene in shielding the atomic QSHI from  oxidation.

## 3 Conclusion

These results mark a significant step forward for ex situ measurements and device fabrication involving intercalated bismuthene. The graphene cap not only protects the material but also opens up opportunities for non‐destructive and cost‐effective quality control methods, such as Raman spectroscopy^[^ ^34^ ^]^ or other optical techniques. This could lead to new insights into exciton physics in bismuthene^[^ ^35^ ^]^ and accelerate progress toward practical applications. Moreover, the demonstrated resistance to oxidation paves the way for future transport measurements, though the conductive graphene layer and substrate doping still presents technological challenges for direct transport studies. In this context, electronic passivation of graphene with, e.g., hydrogen,^[^ ^36^ ^]^ and the use of undoped substrates could address this problem. As compared to our previous work on the QSHI indenene,^[^ ^21^ ^]^ bismuthene's significantly larger topological gap of 800 meV makes such efforts even more promising, bringing room‐temperature quantum spin Hall devices based on atomic monolayers within realistic reach.

## 4 Experimental Section

### Substrate Preparation

Atomically flat N‐doped 4H‐SiC substrates with a resistivity of (0.015 − 0.028) Ωcm and dimensions 12 mm × 2.5 mm were prepared by a dry‐etching technique that saturates the silicon dangling bonds with hydrogen and stabilizes the (1 × 1) SiC(0001) surface.^[^ ^25^ ^]^ The bismuthene intercalation process is detailed in the main text as part of our key findings.

### ARPES and XPS Measurements

Both were performed in our home‐lab setup equipped with a hemispherical analyzer (PHOIBOS 100), a He‐VUV lamp (UVS 300: Figure 4, µSIRIUS: Figure 3), an unmonochromatized Al K‐α light source, and a 6‐axis manipulator capable of LHe‐cooling. ARPES and XPS data shown in Figure 4 (Figure 3, Figure 6) were recorded at 50 K (RT) and a base pressure of <10^−9^ mbar. XPS data shown in Figure 6 are corrected by subtraction of a Shirley background.

### STM Measurements

STM data were recorded at 4.7 K and a base pressure lower than 5· 10^−11^ mbar (Omicron low‐temperature LT STM) using a chemically etched W‐tip that was characterized by imaging the Ag(111) surface state.

### STEM Measurements

Cross‐sectional lamella for scanning transmission electron microscopy (STEM) investigations were prepared on a Dual‐Beam System (FEI Helios Nanolab). Lamella preparation starts with the beam‐induced deposition of a Pt ridge to protect the surface. After Ga ion beam milling, the lamella is lifted out using an Omniprobe micromanipulator and attached to a Cu transmission electron microscope (TEM) grid. The lamella is then thinned to electron transparency using several polishing steps first at 30 keV and finally at 2 keV ion energy. Transfer to the TEM was carried out *ex situ*, exposing the lamella to ambient air for several minutes.

STEM measurements were taken in an uncorrected FEI Titan 80–300 microscope operating at 300 kV acceleration voltage, a beam current of 100–120 pA, a convergence semiangle of 10 mrad and dwell time of 10–20 µs per pixel. The STEM image in Figure 1c were taken in high angle annular dark field (HAADF) mode (scattering angles between 40 and 250 mrad). The spatial resolution under this condition is on the order of 140 pm.

## Remarks

During the review process, we became aware of a preprint reporting bismuthene intercalation results that are consistent with ours.^[^ ^37^ ^]^

## Conflict of Interest

The authors declare no competing interests.

## Author Contributions

L.G. has realized with the help of C.S. and J.E. the epitaxial growth and surface characterization and carried out the photoelectron spectroscopy experiments and their analysis. L.G. and B.L. realized the STM measurements. T.W. performed the oxidation study for pristine bismuthene. On the experimental side, M.K. contributed the TEM images. R.C. and S.M. supervised this joint project.

## Supporting information

Supporting Information

## Data Availability

The data that support the findings of this study are available from the corresponding author upon reasonable request.
