# Supplementary material for: Bismuthene Under Cover: Graphene Intercalation of a Large Gap Quantum Spin Hall Insulator
Source: Adv Mater. 2025 May 20;37(33):2502412. doi: 10.1002/adma.202502412 (PMC12369676; doi:10.1002/adma.202502412)
Supplement: Supplementary file 1 — Supporting Information [file ADMA-37-2502412-s001.pdf]

ADVANCED MATERIALS

Supporting Information

for *Adv. Mater.*, DOI 10.1002/adma.202502412

Bismuthene Under Cover: Graphene Intercalation of a Large Gap Quantum Spin Hall Insulator

*Lukas Gehrig, Cedric Schmitt, Jonas Erhardt, Bing Liu, Tim Wagner, Martin Kamp, Simon Moser and Ralph Claessen**

Supporting Information: Bismuthene Under Cover: Graphene Intercalation of a Large Gap Quantum Spin Hall Insulator

Lukas Gehrig,[1,2] Cedric Schmitt,[1,2] Jonas Erhardt,[1,2] Bing Liu,[1,2] Tim Wagner,[1,2] Martin Kamp,[1,3] Simon Moser,[1,2] and Ralph Claessen[1,2,*]

[1] *Physikalisches Institut, Universität Würzburg, D-97074 Würzburg, Germany*
[2] *Würzburg-Dresden Cluster of Excellence ct.qmat, Universität Würzburg, D-97074 Würzburg, Germany*
[3] *Physikalisches Institut and Röntgen Center for Complex Material Systems, D-97074 Würzburg, Germany*

(Dated: May 16, 2025)

CONTENTS

I. The role of hydrogen in graphene intercalation of bismuthene 2

II. ARPES after air exposure 4

* e-mail: claessen@physik.uni-wuerzburg.de

I. THE ROLE OF HYDROGEN IN GRAPHENE INTERCALATION OF BISMUTHENE

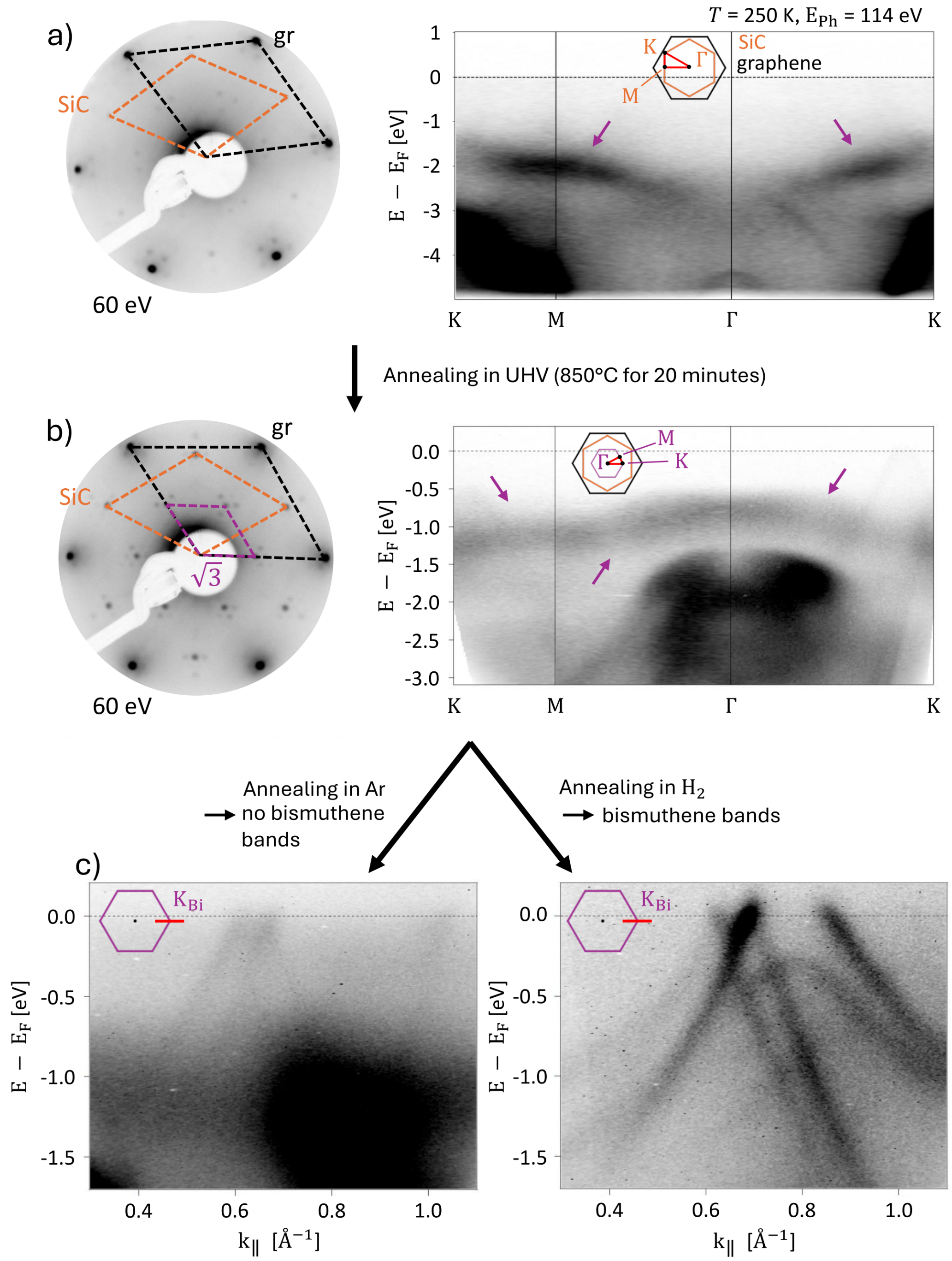

Figure S 1. **The role of hydrogen in the graphene intercalation of bismuthene**. **a)** LEED and ARPES data of the bismuth α phase, exhibiting bands (purple arrows) of a (1×1) periodicity. ARPES data were taken at the MAESTRO 7.0.2 beamline of the Advanced Light Source (ALS). **b)** LEED and ARPES data of the bismuth β phase, exhibiting bands (purple arrows) of a $(\sqrt{3} \times \sqrt{3})R30^\circ$ periodicity. This bismuth β phase is achieved by a bismuth desorption of the α phase at 850°C for 20 minutes. **c)** ARPES data around bismuthene K point after annealing the β phase in argon and hydrogen, respectively, measured at room temperature with $E_{\mathrm{ph}} = 21.2\,\mathrm{eV}$. In stark contrast to the argon case, the hydrogen treated sample shows pronounced bismuthene valence bands. This highlights the crucial role of hydrogen for the intercalation of bismuthene.

To achieve graphene intercalated bismuthene, we first intercalate a full atomic monolayer of bismuth, the so called α phase. The corresponding LEED and ARPES data are shown in Fig. 1a). Desorption of bismuth by annealing at 850°C for 20 minutes from this α phase results in the 1/3 monolayer β phase, whose corresponding ARPES and LEED data is presented in Fig. 1b). Finally, to obtain intercalated bismuthene, the β phase is annealed in a hydrogen atmosphere. As seen in Fig. 1c), this treatment leads to the formation of pronounced bismuthene valence bands, in stark contrast to samples annealed in argon. This emphasizes the role of hydrogen in saturating the silicon bonds at the center of the bismuthene honeycomb, thereby stabilizing the formation of bismuthene and making it energetically favorable.

II. ARPES AFTER AIR EXPOSURE

To assess the oxidation stability of intercalated bismuthene, ARPES from a sample sitting several days in air are shown in Fig. 2. Data was taken at room temperature with $E_{\mathrm{ph}} = 21.2\,\mathrm{eV}$ photons, after the sample was outgassed at 280°C for 120 minutes. Sharp Rashba-split valence bands of bismuthene are clearly observed. This confirms the protective role of the graphene capping layer, which stabilizes the intercalated bismuthene under ambient conditions.

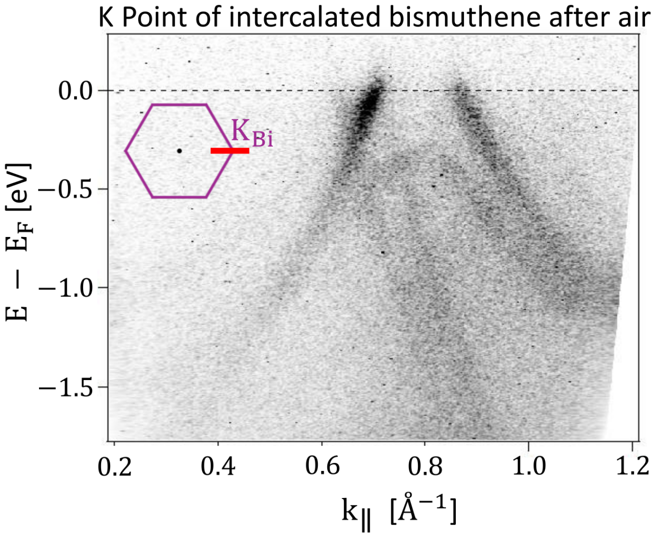

Figure S 2. **Resilience of graphene intercalated bismuthene to air**. ARPES measurement of an intercalated bismuthene sample after exposure to ambient air for several days and a slight outgassing step at 280°C for 120 min, taken at room temperature with $E_{\mathrm{ph}} = 21.2\,\mathrm{eV}$ photons. Both graphene as well as bismuthene valence bands are clearly observable, confirming the oxidation stability of intercalated bismuthene in ambient air.
